# Causes of Hospitalization in Children with Down Syndrome

**DOI:** 10.3390/medicina60091480

**Published:** 2024-09-10

**Authors:** Stefanie Yaemi Takita, Ana Beatriz Silva Sé, Giovanna Michelin Hoffmann, William Bunduki, Lidia Raquel Carvalho, Cátia Regina Branco Fonseca

**Affiliations:** 1 Department of Pediatrics, Botucatu Medical School, São Paulo State University (UNESP), Botucatu 18618-000, São Paulo, Brazil; 2 Department of Biostatistics, Institute of Biosciences, São Paulo University (UNESP), Botucatu 18618-000, São Paulo, Brazil

**Keywords:** Down syndrome, children, hospitalization, health evaluation, outcome assessment, health care

## Abstract

*Background and Objectives*: Down syndrome (DS) is the most common chromosomal disorder in the world. It is caused by the imbalance of the chromosomal constitution of 21 by free trisomy, translocation or mosaicism. Children and adolescents with Down syndrome have immune dysregulation and are more susceptible to infections. This study aims to evaluate hospitalizations of children and adolescents with DS in the pediatric ward of Botucatu Clinics Hospital (HCFMB) and to classify the population of children included in the study according to age, diagnosis, outpatient follow-up, length of stay and need for the intensive care unit (ICU). Thus, it will be possible to improve care for these children, aiming to reduce these hospitalizations. *Materials and Methods*: This study was an observational, cross-sectional study, with retrospective data collected from the last nine years of hospitalization, from January 2013 to December 2021, from children and adolescents with DS in the pediatric ward, emergency room, and the ICU of HCFMB. Children hospitalized in this period in the pediatric ward and ICU, in the age range of 30 days to 15 years, were included in this study. The evaluation of comorbidities that culminated in the need for hospitalization in this population can be the focus of actions to improve the diagnoses and conducts for this population, which can prevent worsening illness and hospitalizations in future populations. *Results*: In this analysis, 80 children with DS were evaluated, with a total of 283 hospitalizations. The most prevalent age group was 1 to 3 years, and the main cause was due to problems in the respiratory system (99 cases). Among the respiratory causes, the main cause of hospitalization was due to pneumonia in 50% of cases, followed by acute respiratory failure in 14%. The average hospitalization time was 8 days, and in 49 hospitalizations, the children required the ICU. The main cause of hospitalization in the ICU was due to respiratory causes (36%), followed by cardiac malformations (14%). During the ICU hospitalizations, there were 13 deaths, and we observed a higher prevalence of heart conditions and, in some cases, positive urine cultures in these children. *Conclusions:* The Hospital serves as a reference for pediatric hospitalizations within its region and beyond, owing to its specialized capabilities. The main causes of hospitalization were those related to the respiratory system and cardiac malformations. Roughly one-third of the children required admission to the intensive care unit.

## 1. Introduction

Down syndrome (DS) is the most common chromosomal abnormality in the population, with an estimated incidence of one in seven hundred live births. The diagnostic hypothesis of DS can be performed in the pre- and postnatal period and confirmed by chromosome analysis, which is referred to as a karyotype test. Children who have the condition are predisposed to have several comorbidities that interfere with their growth and development [1,2,3].

DS is a genetic anomaly characterized by an extra chromosome at position 21 in 92 to 95% of patients. In addition, DS may also be the result of mosaicism in 2 to 4% of patients or translocations in 3 to 4% of cases [1]. According to data from the Brazilian Ministry of Health, a child is born with DS in every 600 to 800 births, regardless of ethnicity, sex or social class [4].

Children and adolescents with DS present with cardiovascular, endocrine, respiratory and musculoskeletal disorders. Patients with DS are more predisposed to autoimmune diseases, including type 1 diabetes mellitus, alopecia areata, vitiligo and celiac disease [5]. Therefore, they should have multidisciplinary and multiprofessional outpatient follow-up.

In children and adolescents with DS, the maturation of T cells in the thymus is aberrant. They exhibit a reduced response to antigens since they produce fewer molecules essential for the recognition of T cell-specific antigens [6]. It is observed that during the early years of life in a child with DS, there is a smaller increase in B and T lymphocytes, suggesting a disturbance of the adaptive immune system [7]. In these children, there is a decrease in the production of active oxygen derivatives, which affects the ability of leukocytes to destroy harmful agents. Consequently, they exhibit immune dysregulation and are more susceptible to infections [8].

Recent decades have seen a substantial increase in the life expectancy of children with DS. In the Netherlands, the infant mortality rate in children with DS dropped from 7.07% in 1992 to 4% in 2003. The fall in DS mortality was mainly related to the successful early surgical treatment of congenital heart defects. The life expectancy of children with DS is primarily dependent on the risk of mortality in the first year of life [9].

Among hospitalizations of these children, according to a study by Soares et al., 50% are due to respiratory problems. This occurs due to the multiple comorbidities associated with DS, such as obstructive sleep apnea syndrome (OSAS), lower airway diseases, congenital heart diseases, relative obesity, hypotonia, pulmonary hypertension, pulmonary hypoplasia, upper airway obstruction and immunodeficiencies, among others. Thus, in addition to being more susceptible to hospitalization, they spend more time in the hospital environment and require ventilatory support disproportionate to the disease [10].

From 2020 to 2022, Brazil and the world experienced the COVID-19 pandemic caused by the SARS-CoV-2 virus [11]. Children with DS exhibit markers of chronic autoimmunity, including high levels of pro-inflammatory cytokines and chemokines. The additional comorbidities found in these children, such as congenital heart disease and obstructive sleep apnea syndrome (OSAS), combined with this pro-inflammatory condition, may exacerbate the infection by the SARS-CoV-2 virus. Therefore, the pediatric community suggests that these children should be more closely monitored due to their increased risk [12]. Treatment flowcharts have been proposed for children with DS and COVID-19, including those proposed by Bambino Gesù Children’s Hospital in Norway, indicating the need for hospitalization or otherwise [13].

The Botucatu Clinical Hospital (HCFMB), in Botucatu city, São Paulo State/Brazil, which provides comprehensive care to patients of the Unified Health System (SUS), is an autarchic entity linked to the São Paulo State Department of Health. It serves 68 municipalities within the Regional Health Directorate (DRS VI)—Bauru. The HCFMB’s service area is estimated to encompass 2 million people. The pediatric ward and the pediatric intensive care unit (PICU) are responsible for inpatient beds and cater to infants, children, and adolescents.

Since 2008, there has been a genetic pediatrics service (SPG) at HCFMB, which provides outpatient follow-up for children and adolescents with DS, thus structuring care for these patients, including karyotype tests for diagnostic confirmation and enabling longitudinal monitoring before and after the hospitalizations considered in this manuscript.

Medical audit records are an instrument for evaluating health care delivery processes due to the ease of access to records, especially medical care [14], and can be used as a source of data, as proposed in this study.

Assessing is therefore a relevant and necessary step in health care. The methods proposed for evaluation are related to its purposes and will have a direct influence on its results [15]. Therefore, this evaluation study impacts inpatient health service, with consequences for the evaluation and improvement of the genetic pediatrics service at HCFMB.

The present study aimed to identify the main causes of hospitalization in children and adolescents with DS at the Botucatu Clinical Hospital over the past nine years, aiming to identify gaps in care that could be improved to prevent some of these admissions and to provide comprehensive care for children with DS.

## 2. Materials and Methods

This study was an observational, cross-sectional study, with retrospective data collected from the last nine years of hospitalization, from January 2013 to December 2021, from children and adolescents with DS in the pediatric wards of HCFMB and other sectors, including the emergency room (ER) and pediatric ICU (PICU). The choice of the study period was because the MV system, the electronic patient record of HCFMB (PEP-MV), was fully implemented in the second semester of 2012, thus enabling reliable data collection.

The local Research Ethics Committee approved the study under protocol CAAE no. 47934121.2.0000.5411, and exception from informed consent was obtained.

### 2.1. Eligibility Criteria and Sample Size

All hospitalizations of children and adolescents diagnosed with DS at any point during care were included, encompassing infants, children and adolescents aged 30 days up to 15 years of age (age of acceptance for admission to this service). Children who do not have DS were excluded.

The consolidated database was provided by a management report in the HCFMB Information System. From PEP-MV records, January 2013 to December 2021, a total of 80 children with DS were hospitalized, of which 46 had more than 1 hospitalization, totaling 283 hospitalizations analyzed. To confirm the diagnosis of DS, the karyotype test, in addition to the information provided by the consolidated database, and its results were searched in the medical records of the electronic medical records.

Diagnoses were sought from the International Disease Code (ICD), 10th Revision (ICD-10, WHO, Geneva, Switzerland), as shown in Table S1 (Supplementary Materials).

From this database, the information considered in the study was as follows: primary care diagnosis according to the International Code of Diseases (ICD), 10th Revision, 2nd edition [16], recorded in care and classified according to the 12 chapters of the Tabulation List of Morbidity for the SUS (ICD-10); total length of stay (in days); and the place of admission—pediatric ward, ICU, or pediatric emergency ward. In a single hospitalization, children can stay in more than one different unit, depending on whether intensive care is needed or not. Other information taken into consideration included the following: patient’s age—classifying them according to age groups, as per classification of the Ministry of Health (SUS), being under one year of age (A), between one and three years of age (B), between four and nine years (C) and greater than or equal to ten years (D) [17]; gender; city of origin—distinguishing between Botucatu and the municipalities within the DRS-VI and the municipalities from other DRS; laboratory tests, including karyotype and requested imaging exams; resources utilized during hospitalization, such as laboratory and imaging tests or surgery; need for oxygen support or need for invasive or non-invasive ventilation in the ICU; death or not during hospitalization.

### 2.2. Statistical Analysis

The database was built directly in an Excel spreadsheet, and statistical analysis was carried out using the Statistical Analysis System (SAS), version 9.2, with descriptive analyses of the studied population extracting means and standard deviations. Chi-square test and Student’s *t* test were used, with a significance level of 5% (*p* < 0.05) [18].

## 3. Results

The average length of stay was eight days, with the shortest period being one and the longest being two hundred fifty-one days, with a median of four days. These children were analyzed according to sex and age group, with 52% being female and a higher incidence of hospitalizations in the age group between one and three years of age. In age group A (under one year old) there were 54 (19%), in B (one to three years old) 130 (46%), in C (four to nine years old) 88 (31%) and in D (over ten years old) 11 children (4%). The origin of the hospitalized children was 143 (50%) from Botucatu, the municipality in which the HCFMB is located, and from other municipalities belonging to the health region (DRS VI), totaling 85%, with the other 15% coming from other regions.

The causes of hospitalization were grouped according to the ICD-10 chapters, with the main cause of hospitalization being those related to the respiratory system, as shown in Figure S1. The main cause of hospitalization was due to diseases of the respiratory system (35%), followed by other infectious diseases (10%), malformations and congenital anomalies (9%) and digestive system diseases (8%).

Among the respiratory system diseases classified by the ICD-10, the most common causes were pneumonia (49 hospitalizations, 50%), followed by acute respiratory failure (14 hospitalizations, 14%). Regarding the ICD-10 of other infectious diseases, the most common causes were unspecified bacterial infections (ten hospitalizations, 35%) and acute diarrhea and gastroenterocolitis (eight hospitalizations, 28%). Among the ICD-10 chapter on malformations, the most prevalent were cardiac malformations (twelve hospitalizations, 44%) and ectopic testicles (five hospitalizations, 18%).

Out of the total of 283 hospitalizations analyzed, 82 (29%) of these were in the intensive care unit (ICU), and these hospitalizations occurred for 62 children. A total of 21 of these hospitalizations were initially admitted to the emergency room, totaling 26% of ICU admissions. In the data analysis, the number of hospitalizations is considered, not the number of children admitted to the ICU.

The resources used during hospitalization in the emergency room, ward or ICU were classified as follows: oxygen support (venturi mask, face mask, O_2_ catheter), use of antibiotics, chemotherapy or other medications and carrying out laboratory and imaging tests, as shown in Figure S2.

### 3.1. Admissions to the Pediatric ICU at HCFMB

The average length of stay in the ICU was 24 days. The main cause of hospitalization was respiratory diseases (23 hospitalizations, 28%), with 12% (10 hospitalizations) due to acute respiratory failure and 16% (13 hospitalizations) due to bacterial pneumonia. The second main cause was cardiac malformation correction (twelve hospitalizations, 14%), with 10% (eight hospitalizations) for atrioventricular septal defect (AVSD) correction and 4% (three hospitalizations) for VSD correction.

Regarding the resources used during ICU admissions, 58 (71%) children had their karyotype recorded, confirming the diagnosis of DS in the hospitalization period. For 22 patients (27%) the karyotype was not available through the consolidated database, but they were present in the patient’s medical record and were carried out at the service. In only two hospitalizations (2.4%), this genetic test was not carried out in the service and must therefore have been carried out outside of the HCFMB, since the DS diagnosis was included in the hospitalization ICD, and the children were being followed-up with at the SPG.

Of these patients, 65% underwent an echocardiogram, an important test in the diagnosis of cardiac malformations.

Regarding ventilatory support, 35 of the hospitalizations required mechanical ventilation and 29 of them were on non-invasive ventilation. It must be considered that the same patient may use more ventilatory support according to their needs; therefore, it is not possible to count this as a percentage (Figure S3).

As shown in Table S2, out of the surgeries performed, ten (23%) were due to ophthalmological causes. HCFMB is one of the few reference services for the treatment of congenital cataracts in the state of Sao Paulo. Of the other surgeries performed, nine (19%) were for correction of cardiac malformations and five (10%) were to correct otorhinolaryngological causes in the DS population.

An unfavorable outcome of hospitalizations is death, which was recorded for 13 of the 62 children (21%) admitted to the ICU.

### 3.2. Characterization of the Children Who Died

The average age of children who died was 7.8 months (SD = 6.6), with no statistically significant difference with those who did not die (*p* = 0.37). The median was 7 months, with a maximum age of death being 21 months (1 year and 9 months).

The most prevalent comorbidities in these patients were cardiac malformations, followed by thyroid, ophthalmic, pulmonary and gastrointestinal tract diseases (Table S3). The average length of stay was 33 days, with a median of 10 days, a minimum of 2 days and a maximum of 224 days.

There was a statistically significant relationship between deaths and urine culture results (Table S4), with four children who died having positive urine cultures. Blood culture results were not statistically significant.

In the analysis of the distribution of admissions and deaths of children admitted to the pediatric ICU, according to the year of occurrence, a higher concentration of these two events can be observed in the years 2019 to 2021, with 34 ICU admissions (41.5% in three years), and 20.6% of deaths (n = 7), versus 48 admissions between 2013 and 2018 (58.5% in six years) with six deaths (12.5%) (*p* = 0.82).

## 4. Discussion

Patients admitted with DS totaled 283 hospitalizations in this study, with 29% of them (82 hospitalizations) requiring intensive care in the ICU. Considering the resources used during hospitalization, 35 of them required mechanical ventilation. Considering that children with DS have other associated comorbidities, such as cardiac malformations and endocrinological dysfunctions, they require greater attention in their care, often requiring intensive care [19], as shown by the present study.

It was found that, regardless of age group, the main causes of hospitalizations were respiratory diseases (5.8 million, 21% of hospitalizations), as seen in this study. In the Ministry’s analysis, it is also possible to verify that the age group with the highest number of hospitalizations was those under four years of age, as was also proven in this work. On the other hand, deaths, according to the SIH/SUS, were more prevalent in children under one year of age (55%), mainly due to conditions originating in the perinatal period. In this study, the mean age at death was seven months, and comorbidities associated with DS should be considered as responsible for early death in these children [1,2]. This is incompatible with the current technologies for monitoring and treating them in Brazil, as this scenario has been undergoing transformations and advances in medicine, especially genetics and cardiology, which have contributed to substantially changing the statistics. Specialists linked to the Brazilian Society of Cardiology (SBC) affirm that, regarding prognosis, the survival of these children has significantly increased in the last three decades [20].

Among the comorbidities that justified hospitalization for surgical intervention, congenital cataracts should be highlighted. This requires a surgical approach and there is an association between cataracts and strabismus and nystagmus in children with DS, and its correction is important in order to enable advances in the adequate development of these children. Furthermore, due to the greater risk of developing post-surgical complications, such as capsular opacification and retinal detachment [21,22,23], it is justified to monitored this at cataract reference centers such as the HCFMB.

According to the literature, children with DS may have multiple organs and systems compromised. However, the most associated congenital malformation is of cardiac origin, with 40% to 60% of individuals with DS affected [24]. It is the main cause of morbidity and mortality in the first 2 years of these children’s lives [25]. Those with congenital heart disease have low weight gain, with longer ICU stay. Furthermore, female sex is associated with congenital heart disease and is related to a higher risk of death in hospitals [26].

Cardiac malformation was the main comorbidity associated with death, and it was the second most prevalent cause of ICU admission. Analyzing the hospitalizations of children and adolescents with congenital heart diseases, it is possible to verify the predominance of those belonging to families with unfavorable socioeconomic conditions, which corroborates the need for public policies to promote health for these children [27]. An echocardiogram is an important test in the diagnosis of cardiac malformations. However, only 65% of patients admitted in this study underwent it. Furthermore, 58 children (71%) had the diagnosis of DS confirmed by karyotype during the hospitalization. This insufficient number of tests collected by the consolidated database demonstrates the importance of adequate registration in the information system, which is essential for assistance and also for research. Given this fact, the search for diagnostic tests led to a survey of medical records, resulting in only two children (2.4%) that did not have a karyotype test performed at HCFMB.

As observed in this study and confirmed by the literature, the main cause of hospitalization in children with DS, and also in those with congenital heart disease, is due to respiratory diseases [28]. Therefore, these patients may present bronchopneumonia and progress to acute respiratory failure, corroborating the main ICD-10 hospitalizations in all sectors of pediatrics at HCFMB in the period from 2013 to 2021. While modern medical care has reduced the mortality rate to more acceptable values, both morbidity and mortality could be further reduced. In this respect, respiratory infections and neonatal problems are the most important issues to be solved [9].

Considering this, one of the interventions to prevent new hospitalizations is vaccination. The 23-valent pneumococcal vaccine is recommended for children with DS by the Brazilian Society of Immunization and the Brazilian Society of Pediatrics [1,2], bringing scientifically proven benefits to them [29]. Palivizumab in Brazil is currently indicated for children born prematurely with bronchopulmonary dysplasia and/or congenital heart disease. The adoption of this measure could reduce the inconvenience caused by RSV during seasonal periods in this population [30].

In the present study, it was seen that from the years 2019 to 2021, there was a proportionally higher percentage of ICU admissions as well as deaths in comparison to the previous six years, suggesting a temporal correlation with the COVID-19 pandemic, with some studies finding a relationship between DS and greater severity and complications such as sepsis, as well as a relationship between the need for mechanical ventilation and severe acute respiratory distress syndrome (SARS) with COVID-19 [31,32]. Therefore, COVID-19 is also associated with high mortality in children with DS, even after adjusting the analysis for sociodemographic factors and comorbidities such as cardiovascular diseases present in this population [33,34].

Deaths in pediatrics can occur due to various etiologies, including infections. In a study on neonatal deaths occurring at a hospital similar to HCFMB, 66% (207 children) had at least one positive microbiological culture, isolating micro-organisms in tracheal secretions, swabs, blood, urine and liquor [35]. One of the most important complications of cardiac surgery in patients with DS is postoperative infection, mainly associated with low weight and inadequate antibiotic prophylaxis [36]. The mortality percentage of children with DS was high for the years of the study, as well as for the technology present in the reference hospital, when compared to those in the Netherlands, even in 1992 [9].

Children with sepsis and DS have a high risk of death, even after adjusting for potentially confounding factors, such as demographics, pathogens and concomitant conditions [37]. As observed in this study, a statistically significant correlation was found between positive urine culture and death.

The limitations of this study include a reliance on a consolidated report rather than individual medical records, resulting in the absence of specific diagnoses for sepsis, septic shock and COVID-19 confirmation. Additionally, none of the ICD-10 diagnostic codes listed in the corresponding chapters precisely matched these conditions. The use of consolidated databases can be limiting when records are incomplete, despite providing large amounts of information in a quick time.

The strength of this study was that it began studying these hospitalizations and sought data so that health promotion and disease prevention actions could be improved in the outpatient follow-up of these children.

Based on the findings of this study, a proposal for a risk score for death in children and adolescents with DS who were admitted to the ICU was elaborated (Table S5). The cut-off value for age was according to the group with the highest number of hospitalizations and deaths in our study (under 2 years of age). A statistically significant relationship was seen between urine culture and deaths. It must be considered that a culture is collected in the most seriously ill patients. Therefore, even if it is negative, its performance is an important piece of data to be considered in the score. The most prevalent comorbidity in deaths in this study was cardiac malformation, which is why it is considered for a higher score. For length of stay, the median of the present study (10 days) was considered.

According to this scoring system, patients who score six points or more are at a higher risk of mortality and should be monitored more closely.

The risk score proposal still needs to be validated in future studies. And, it may also include items related to preventive measures, such as vaccinations and adequate outpatient follow-up of these children.

## 5. Conclusions

The Hospital of this study serves as a reference for pediatric hospitalizations within its region and beyond, owing to its status as a tertiary hospital with specialized capabilities.

The main causes of hospitalization for children with DS were those related to the respiratory system. Roughly one-third of these children required admission to an intensive care unit, with a high prevalence of cardiac malformations.

## Data Availability

The data presented in this study are available on request from the corresponding author. The data are not publicly available due to ethical restrictions.

## Supplementary Materials

The following supporting information can be downloaded at https://www.mdpi.com/article/10.3390/medicina60091480/s1, Figure S1: Disease Code (ICD) for Down Syndrome.; Figure S2: Distribution of resources utilized during hospitalization from 2013 to 2021; Figure S3: Ventilatory support utilized by DS patients during admissions in ICU, from 2013 to 2021; Table S1: International Disease Code (ICD) for Down Syndrome.; Table S2: Distribution of the types of surgeries performed during hospitalization.; Table S3: Frequency distribution of the 82 admissions to the ICU, according to type of comorbidities and death.; Table S4: Distribution of culture results from children with DS admitted to the ICU and who died.; Table S5: Proposal of risk score for death in children with Down syndrome admitted to the ICU, Botucatu, 2023.
