# Supplementary material for: Causes of Hospitalization in Children with Down Syndrome
Source: Medicina (Kaunas). 2024 Sep 10;60(9):1480. doi: 10.3390/medicina60091480 (PMC11433946; doi:10.3390/medicina60091480)
Supplement: Supplementary file 1 [file medicina-60-01480-s001.zip › medicina-3123511-supplementary.pdf]

medicina

MDPI

Supplementary Materials

Table S1. International Disease Code (ICD) for Down Syndrome.

**ICD 10 - Q90**	**Down Syndrome**
**ICD** 10 - Q90.0	Trisomy 21, nonmosaicism (meiotic nondisjunction)
**ICD** 10 - Q90.1	Trisomy 21, mosaicism (mitotic nondisjunction)
**ICD** 10 - Q90.2	Trisomy 21, translocation
**ICD** 10 - Q90.9	**Down syndrome**, unspecified

Table S2. Distribution of the types of surgeries performed during hospitalization, 2013-2021 (n=44).

Type of surgery	N	%
Examination of the eye under anesthesia	10	22.7
Posterior capsulectomy surgery	1	2.2
Enterectomy	1	2.2
Correction of ventricular septal defect (VSD)	4	9.1
Surgical treatment of patella fracture by internal fixation (patellectomy)	1	2.2
Tonsillectomy with adenoidectomy	4	9.1
Exploratory laparotomy	4	9.1
Total atrioventricular canal correction	1	2.2
Unilateral orchiopexy	3	6.8
Pulmonary artery banding	2	4.5
Bilateral laparoscopic orchiopexy	1	2.2
Hypospadias correction	1	2.2
Atrioventricular canal defect repair	2	4.5
Removal of a foreign body from the digestive tract by endoscopy	1	2.2
Umbilical hernia repair	1	2.2
Cystoscopy and/or ureteroscopy and/or uteroscopy	1	2.2
Gastrostomy	1	2.2
Surgical treatment of vesicoureteral reflux	1	2..2
Adenoidectomy	1	2.2
Laparoscopic cholecystectomy	1	2.2
Phacoemulsification with implantation of foldable intraocular lenses	1	2.2
Incisional hernia repair	1	2.2

Table S3. Frequency distribution of the 82 admissions to the ICU, according to type of comorbidities and death, 2013-2021 (p=0.60).

**Comorbidity**	**Death**				
	**No**		**Yes**		**Total**
	Amount	%	Amount	%	
None	35	87.5	5	12.5	40
Type 1 diabetes mellitus	1	100.0	0	0.0	1
Cardiac malformation	54	79.4	14	20.6	68
Neurological – epilepsy	1	100.0	0	0.0	1
Ophthalmological – cataract	1	50.0	1	50.0	2
Oncological – Acute Myeloid Leukemia (AML)	1	100.0	0	0.0	1
Musculoskeletal – umbilical hernia	1	100.0	0	0.0	1
Genitourinary	4	100.0	0	0.0	4
Gastrointestinal	8	96.3	1	3.7	9
Thyroid – Hypothyroidism	25	92.6	2	7.4	27
Pulmonary	2	66.7	1	33.3	3
Orthopedic – congenital clubfoot	1	100.0	0	0.0	1
Otorhinolaryngological	4	100.0	0	0.0	4
**Total**	69	84.2	13	15.8	82

Table S4. Distribution of culture results from children with DS admitted to the ICU and who died, 2013-2021.

Culture	Death				Total	*P*
	No		Yes			
	Amount	%	Amount	%	Amount	
<u>URINE</u>						
Negative	57	87.7	8	12.3	65	
Positive	12	70.6	5*	29.4	17	**0.03**
<u>BLOOD</u>						
Negative	47	87.0	7	13.0	54	
Positive	22	78.6	6**	21.4	28	0.61
**Total**	**69**	**84.1**	**13**	**15.9%**	**82**	

** Candida spp was present in four children (29,5%); ** Trichosporon asahii was present in one child (3,6%).*

Table S5. Proposal of risk score for death in children with Down syndrome admitted to the ICU, Botucatu, 2023.

INFORMATION	2 points	1 point	0 point
**Age**	0-3 years	4-9 years	>10 years
**Comorbidity**	Cardiac malformation	One or more comorbidities, except cardiac malformation	No comorbidities
**Ventilatory support**	Mechanical ventilation	High-flow nasal cannula<br>Non-invasive ventilation<br>Oxygen supplementation	Without ventilatory support
**Outpatient follow-up**	No follow-up	Intermittent follow-up	Regular follow-up
**ICU length of stay**	10 days or more	1-9 days	0 day

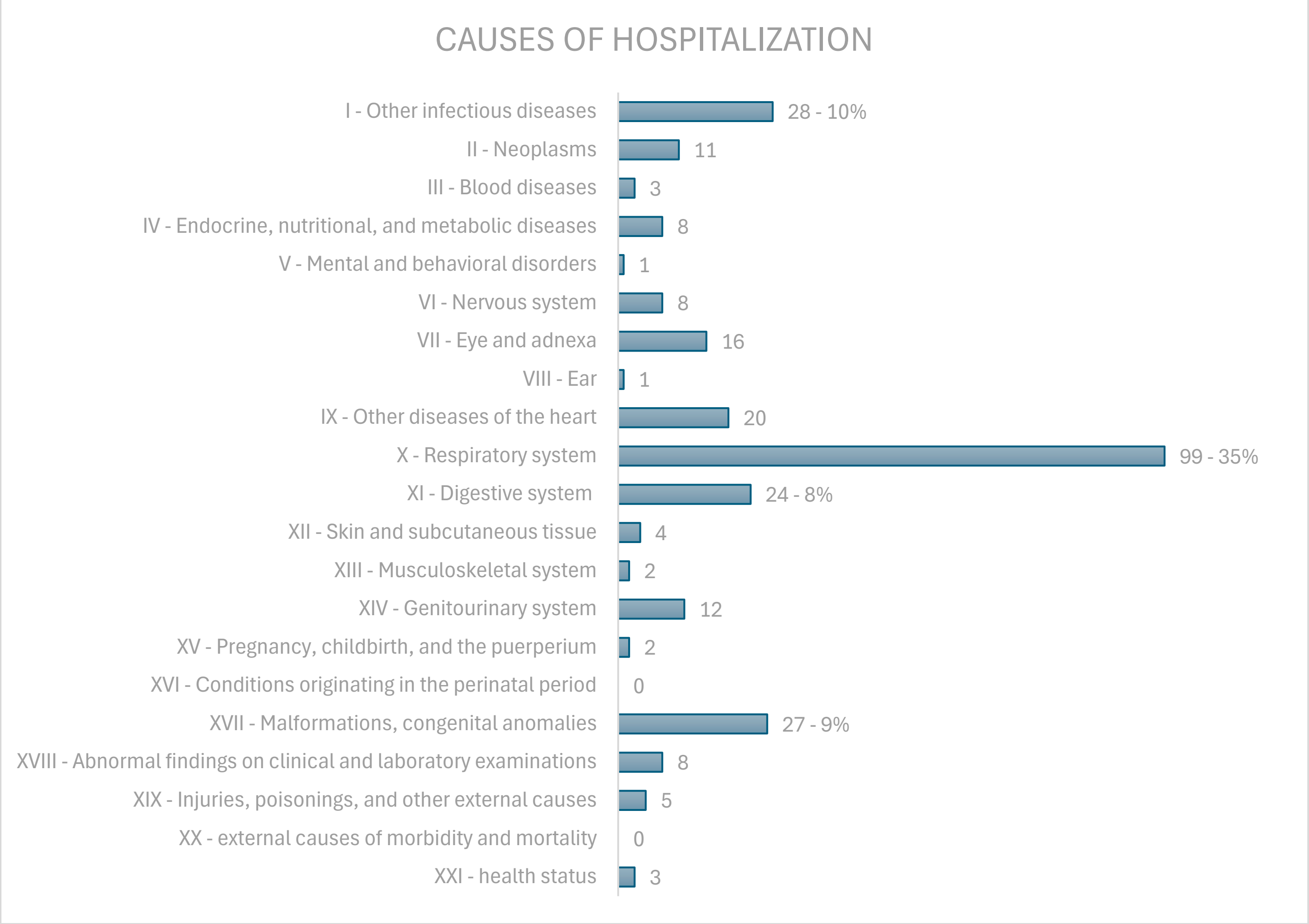

Figure S1. Diagnoses upon admission and classified according to the ICD-10 chapters.

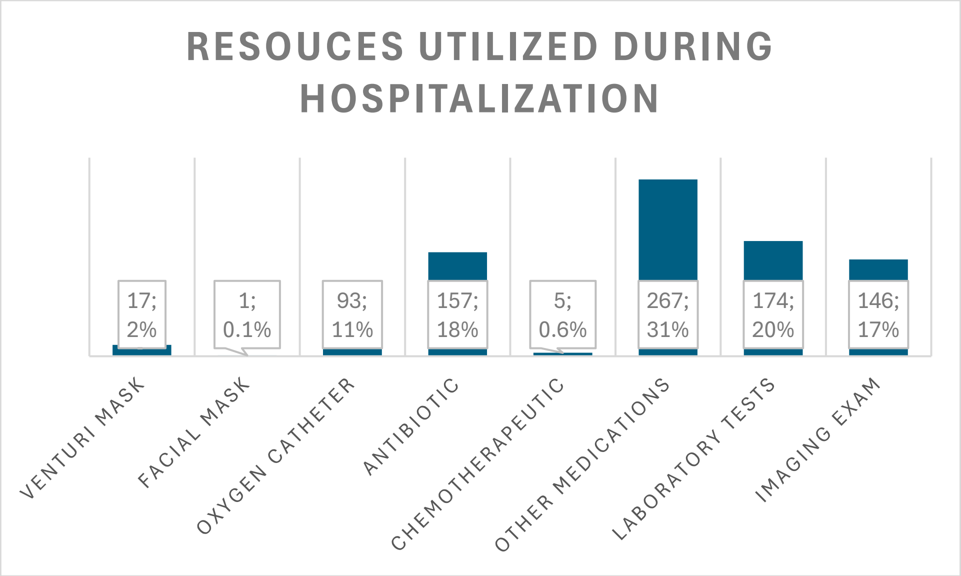

Figure S2. Distribution of resources utilized during hospitalization from 2013 to 2021. Percentage calculated over the total resources utilized in hospitalizations of patients with Down syndrome during the described period (total: 860).

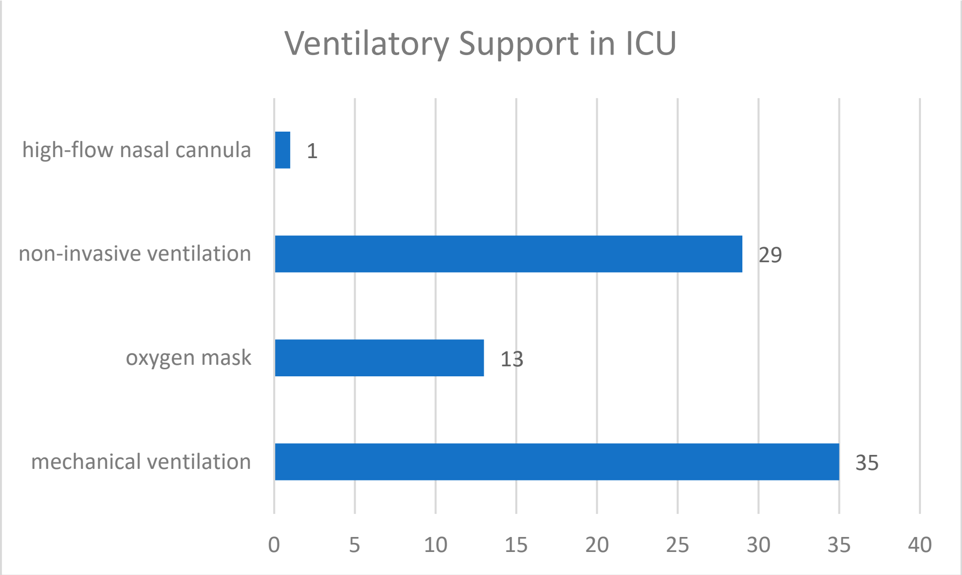

Figure S3. Ventilatory support utilized by DS patients during admissions in ICU, from 2013 to 2021.
